# Agreement, repeatability, and reproducibility of quantitative retinal layer assessment using swept-source and spectral-domain optical coherence tomography in eyes with retinal diseases

**DOI:** 10.3389/fmed.2023.1281751

**Published:** 2023-12-18

**Authors:** Huiyuan Hou, Mary K. Durbin, Nevin El-Nimri, Jeffrey L. Fischer, Srinivas R. Sadda

**Affiliations:** ^1^Topcon Healthcare, Oakland, NJ, United States; ^2^Fischer Laser Eye Center, Willmar, MN, United States; ^3^Doheny Eye Institute, Pasadena, CA, United States; ^4^Department of Ophthalmology, David Geffen School of Medicine, University of California, Los Angeles, Los Angeles, CA, United States

**Keywords:** optical coherence tomography, repeatability, reproducibility, agreement, retinopathy

## Abstract

**Purpose:**

To evaluate the agreement and precision of retinal thickness measurements obtained using swept-source optical coherence tomography (SS-OCT) and spectral-domain OCT (SD-OCT) in healthy eyes and eyes with retinopathy.

**Methods:**

This cross-sectional prospective study involved three DRI-OCT Triton (SS-OCT) and three 3D-OCT-1 Maestro (SD-OCT) devices. One of each device (Maestro and Triton) was paired with a single operator. Healthy subjects and patients with retinal diseases were recruited, with study eye and testing order randomized. At least 3 scans per eye were captured for wide scan (12 mm × 9 mm-Triton and Maestro) and macular cube scan (7 mm × 7 mm-Triton, 6 mm × 6 mm-Maestro). Thickness of the full retina, ganglion cell layer + inner plexiform layer (GCL+), and ganglion cell complex (GCL++) were obtained from wide scan and cube scans. Agreement of the measurements between the Triton and Maestro was evaluated by Bland–Altman analysis and Deming regression for each group. Repeatability and reproducibility were assessed using a two-way random effect analysis of variance (ANOVA) model for each parameter by group.

**Results:**

Twenty-five healthy subjects (25 eyes) and 26 patients with retinal diseases (26 eyes), including, but not limited to, age-related macular degeneration, macular hole, and diabetic retinopathy were recruited. Overall, the measurement differences between Triton and Maestro were <6 μm (mean differences of full retina, GCL++, and GCL+ thickness were ≤5.5 μm, 1.3 μm, and 2.8 μm, respectively) and not statistically significant across the parameters. The repeatability and reproducibility estimates indicate high precision in both devices and groups. Across all the parameters, the repeatability limit was ≤7.6 μm for Triton and ≤12.7 μm for Maestro; reproducibility limit was ≤9.2 μm for Triton and ≤14.4 μm for Maestro. In eyes with retinal pathology, the repeatability coefficient of variation (CV)% was ≤2.6% for Triton and ≤3.4% for Maestro; reproducibility CV% was ≤3.3% for Triton and ≤3.5% for Maestro.

**Conclusion:**

Both Triton SS-OCT and Maestro SD-OCT provide reliable measurements of retinal thickness in healthy eyes and eyes with retinal diseases. Excellent agreement between the two devices indicates interoperability when testing healthy eyes or eyes with retinal pathology. These findings support the use of thickness measurements from Triton SS-OCT and Maestro SD-OCT in clinical practice.

## Introduction

Optical coherence tomography (OCT) is an indispensable imaging technology that enables the capture of *in vivo*, non-invasive images of the retina and choroid (1). OCT technology has greatly improved over the years since it was first introduced. The development of Fourier domain OCT has facilitated improving both resolution and speed over time-domain OCT (2, 3). The two types of Fourier domain detection are spectral-domain OCT (SD-OCT) and swept-source domain OCT (SS-OCT) (3). SS-OCT devices generally offer higher scanning speed than SD-OCT (4, 5) and utilize longer wavelengths, allowing better visualization of deep structures such as the choroid, lamina cribrosa, and sclera (6, 7). The high-speed wavelength tuning laser known as swept source, digital data acquisition and processing technology enable SS-OCT to mitigate the signal roll-off observed for SD-OCT (7). The longer wavelength light source also provides greater penetration that renders SS-OCT optimal for imaging eyes with media opacity (7). On the other hand, because axial resolution scales with the square of the central wavelength and the inverse of the bandwidth of the applied light source, SD-OCT devices typically have higher axial resolution than SS-OCT (6). As the two main commercially available OCT systems, each technology has distinct benefits. To facilitate their clinical application and optimization, it is important to thoroughly compare measurements obtained with SS-OCT and SD-OCT systems.

Volumetric scan images and automated retinal thickness measurements generated by the OCT systems are used clinically to qualitatively and quantitatively evaluate the different layers of the retina, facilitating the diagnosis and monitoring of various ocular diseases such as age-related macular degeneration (AMD) and macular edema from various causes (1, 8, 9). It is now known that many retinal disorders that initially affect the outer retina can lead to subsequent alterations in the inner retina including the retinal ganglion cell (RGC) layer; moreover, degenerative loss of inner retinal layers can occur in early diabetic retinopathy (10). These have resulted in increasing interest in the quantitative assessment of the inner retinal layers, separately from the overall retinal thickness (11). Before one can confidently use measurements from either SS-OCT or SD-OCT in clinical trials or clinical practice, establishing repeatability and reproducibility is critical to judge whether differences between individuals or changes in these measurements over time are of significance (12). Repeatability establishes the variability expected in a measurement over time if nothing changes, while reproducibility considers comparisons of data obtained from different devices and different operators, which might happen in a clinic with multiple instruments, in a multi-site practice, or in the context of multicenter clinical trials (13). A comparison of the repeatability and reproducibility of SS-OCT and SD-OCT as well as an evaluation of the agreement of their measurements would provide essential information regarding their ability to produce reliable examination results and their potential interchangeability in a clinical setting.

The aim of the present study was to evaluate the agreement, repeatability, and reproducibility of thickness measurements of various retinal layers using SS-OCT (DRI OCT Triton, Topcon Inc., Tokyo, Japan) which scans at speeds of 100,000 A scans per second and SD-OCT (3D OCT-1 Maestro, Topcon Inc., Tokyo, Japan) which operates at 50,000 A scans per second, in healthy eyes and eyes with various retinal pathologies.

## Methods

The protocol of this prospective study was approved by the IntegReview Institutional Review Boards (3815 S. Capital of Texas Hwy, Suite 320, Austin, TX 78704), and the methodology adheres to the tenets of the Declaration of Helsinki for research involving human subjects and to the Health Insurance Portability and Accountability Act. The recruitment started on March 13, 2017, and ended on April 26, 2017. Written informed consent was obtained from all subjects.

### Participants

The following ocular examinations were performed on each eye of each subject to determine eligibility for the study: best-corrected visual acuity (BCVA), refraction, slit lamp biomicroscopy, ophthalmoscopy, intraocular pressure (IOP) and visual field (VF; standard automated perimetry, Humphrey Field Analyzer; 24-2 Swedish interactive threshold algorithm; Carl Zeiss Meditec, Inc., Dublin, California).

In order to be included in this study, subjects had to be 18 years of age or older on the date of informed consent, be able to understand the written informed consent, be willing to participate as evidenced by signing the informed consent and had IOP ≤21 mmHg bilaterally. Exclusion criteria included being unable to tolerate ophthalmic imaging, having ocular media that precluded obtaining acceptable OCT images, having narrow angles that would preclude dilation, and having a history of systemic diseases or medications that might affect the measurements, such as leukemia, dementia or multiple sclerosis, or concomitant use of hydroxychloroquine or chloroquine.

Healthy subjects (Healthy group) had BCVA 20/40 or better and normal ocular health bilaterally (non-visually impairing cataract was acceptable). Both eyes must have met all normal eligibility criteria prior to study eye randomization. An additional exclusion criterion for the Healthy group was evidence of VF defects consistent with glaucomatous optic nerve damage based on at least one of the following two findings: a cluster of 3 or more points in an expected location of the pattern deviation (PD) depressed below the 5% level, at least 1 of which was depressed below the 1% level; or having glaucoma hemi-field test flagged on the VF report as being “outside normal limits.”

Subjects with retinal disease (Retina group) were included if they were diagnosed with a retinal pathology including, but not limited to AMD, diabetic macular edema, diabetic retinopathy, macular hole, and epiretinal membrane. Subjects were excluded from the Retina group if they had glaucoma, ocular surface disease, or any ocular pathology other than retinal disease in the study eye (non-visually impairing cataract was acceptable). The eye with the specific pathology was deemed the study eye. If both eyes were eligible, then one eye was randomly selected to be the study eye.

### Optical coherence tomography scans

In this study, 3 SS-OCT devices (DRI OCT Triton, Topcon Inc., Tokyo, Japan) and 3 SD-OCT devices (3D OCT-1 Maestro, Topcon Inc., Tokyo, Japan) were employed. Three operators were paired with one Triton and one Maestro each to create three distinct operator/device configurations. Eligible subjects were randomized to select the testing order of the operator/device configuration. All OCT imaging was performed in one session, with breaks at the discretion of the subject. The scan types included wide scan (12 mm × 9 mm-both devices) and macular cube scan (7 mm × 7 mm-Triton, 6 mm × 6 mm-Maestro). For each scan type, a minimum of 3 scans was performed per eye for each configuration of operator/device. Additional scans were acquired by the operator if the scan quality was determined to be unacceptable. Parameter measurements included thickness of the full retina in nine sectors as defined by the early treatment of diabetic retinopathy study (ETDRS), ganglion cell layer (GCL) + inner plexiform layer (IPL) (GCIPL/abbreviated on the instrument reports and in this study to GCL+), and ganglion cell complex (GCC/abbreviated on the instrument reports and in this study to GCL++).

Two imaging experts reviewed each scan independently for image quality acceptance in a randomized fashion. Key aspects of a scan to consider for acceptable image quality were: (1) overall signal strength, (2) local weak signal, (3) poor centration of key structures (fovea not in center of macula scans), (4) eye movements, (5) clipping of the retina (scan is too high or too low and the full retina is chopped off), (6) segmentation failure, and (7) improper placement of the macula grid. Acceptable quality scan passed each of these image quality tests. All scans deemed unacceptable were not included in the data analysis.

### Statistical analysis

Continuous variables were analyzed using descriptive statistics, including total number (*n*), mean, standard deviation (SD), and median, and categorical variables were summarized using percentages.

Agreement analysis of OCT B-scan image quality between the Triton and Maestro was performed by group. Cross-tabulation of the grading results between the study devices was provided by grader and group.

The first acceptable scan from each scan type (wide scan and macular cube scan) from the Triton and Maestro was used for agreement analyses. Bland–Altman analysis was used to calculate the mean difference and limits of agreement (LOA), and Deming regression was used to calculate the intercept and slope for linear fitting model.

All acceptable scans from the Triton and Maestro were used in the precision analysis, which was based on a two-way random effect analysis of variance (ANOVA) model. This ANOVA model included the operator/device, eye, and interaction between operator/device and eye as variance components. The repeatability and reproducibility limits and coefficient of variation in percentage (CV%) were calculated as follows: repeatability SD = square root of the residual variance; reproducibility SD = square root of the sum of the operator/device variance, the interaction variance, and the residual variance; repeatability limit = 2.8 x repeatability SD; reproducibility limit = 2.8 × reproducibility SD; repeatability CV% = (repeatability SD)/intercept × 100%; reproducibility CV% = (reproducibility SD)/intercept × 100%.

The sample size was determined based on the 95% LOA and the two-way random effect ANOVA model for precision. To acquire at least 90% power at a one-sided significance level of 5% using an *F*-test to detect a variance of operator/device effect that was 50% of the total variance, it was determined that a sample size of 21 eyes per population was sufficient. Statistical software SAS 9.3 (SAS Institute, Cary, North Carolina) was used for all calculations. *p*-values less than 0.05 were considered statistically significant.

## Results

Eleven subjects did not meet the eligibility criteria and one subject withdrew consent. Twenty-five healthy subjects (25 eyes) and 26 retinal disease subjects (26 eyes) were included. Demographic and ocular characteristics of the study subjects are summarized in Table 1. The Retina group was older in age and had worse BCVA than the Healthy group. Retinal disease diagnoses included macular degeneration (*n* = 10), epiretinal membrane (*n* = 12), macular hole (*n* = 3), cystoid macular edema (n = 3), diabetic retinopathy (n = 1), and other retinal diseases (*n* = 15). The Retina group study eyes may have had more than one diagnosis.

**Table 1:** Demographics and ocular characteristics of study subjects.

	Healthy	Retina
**By subject (No.)**	25	26
Age (years)	42.7 ± 14.7	67.2 ± 11.3
*Age group, No. (%)*		
<65 years	24 (96)	11 (42)
≥65 years	1 (4)	15 (58)
Gender (M/F)	14/11	9/17
Race, Caucasian No. (%)	25 (100)	26 (100)
**By Eye (No.)**	25	26
*BCVA, No. (%)*		
20/20 or better	24 (96)	11 (42)
20/21-20/40	1 (4)	15 (58)
MRSE (D)	−0.70 ± 1.61	−0.48 ± 1.73
Axial length (mm)	23.93 ± 1.08	23.87 ± 1.07
IOP (mmHg)	15.2 ± 3.1	15.3 ± 3.9

Continuous data are shown as mean ± standard deviation. BCVA, best corrected visual acuity; D, diopter; MRSE, manifest refractive spherical equivalent; IOP, intraocular pressure.

Overall, good agreement of B scan image quality between the Triton SS-OCT and Maestro SD-OCT was found.

In general, Bland–Altman analysis showed that the measurement differences between Triton and Maestro were less than 6 μm across the parameters. Deming regression showed most of the slopes of the fitting model between Triton and Maestro were close to +1, and most of the 95% confidence intervals (CI) for intercept contained 0 and CI for slope contained 1.

**Figure 1:**
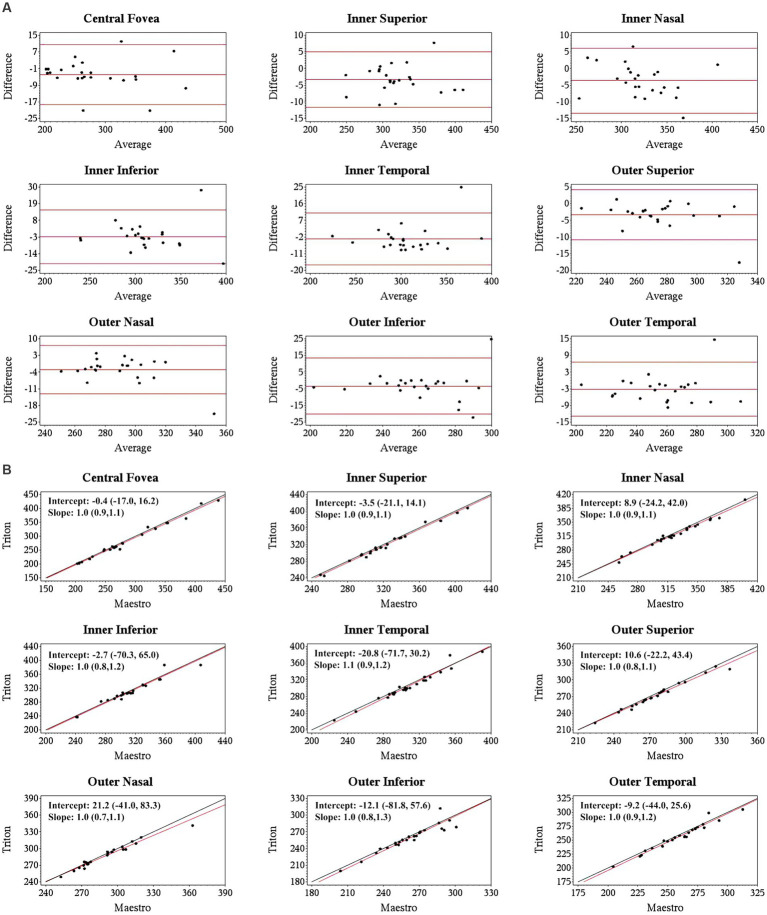
Agreement of full retina thickness measurements from the wide scan between Triton SS-OCT and Maestro SD-OCT in eyes with retinal disease. **(A)** Bland–Altman plots show all the average measurement differences between the two devices are less than 4 μm. **(B)** Deming regression plots of full retina thickness from the wide scan. The plots illustrate the fitted linear models (red line) and the identity lines (Triton measurement = Maestro measurement, slope = 1) (black line). Intercepts and slops are shown as mean (95% confidence interval). All slopes are very close to +1.

**Figure 2:**
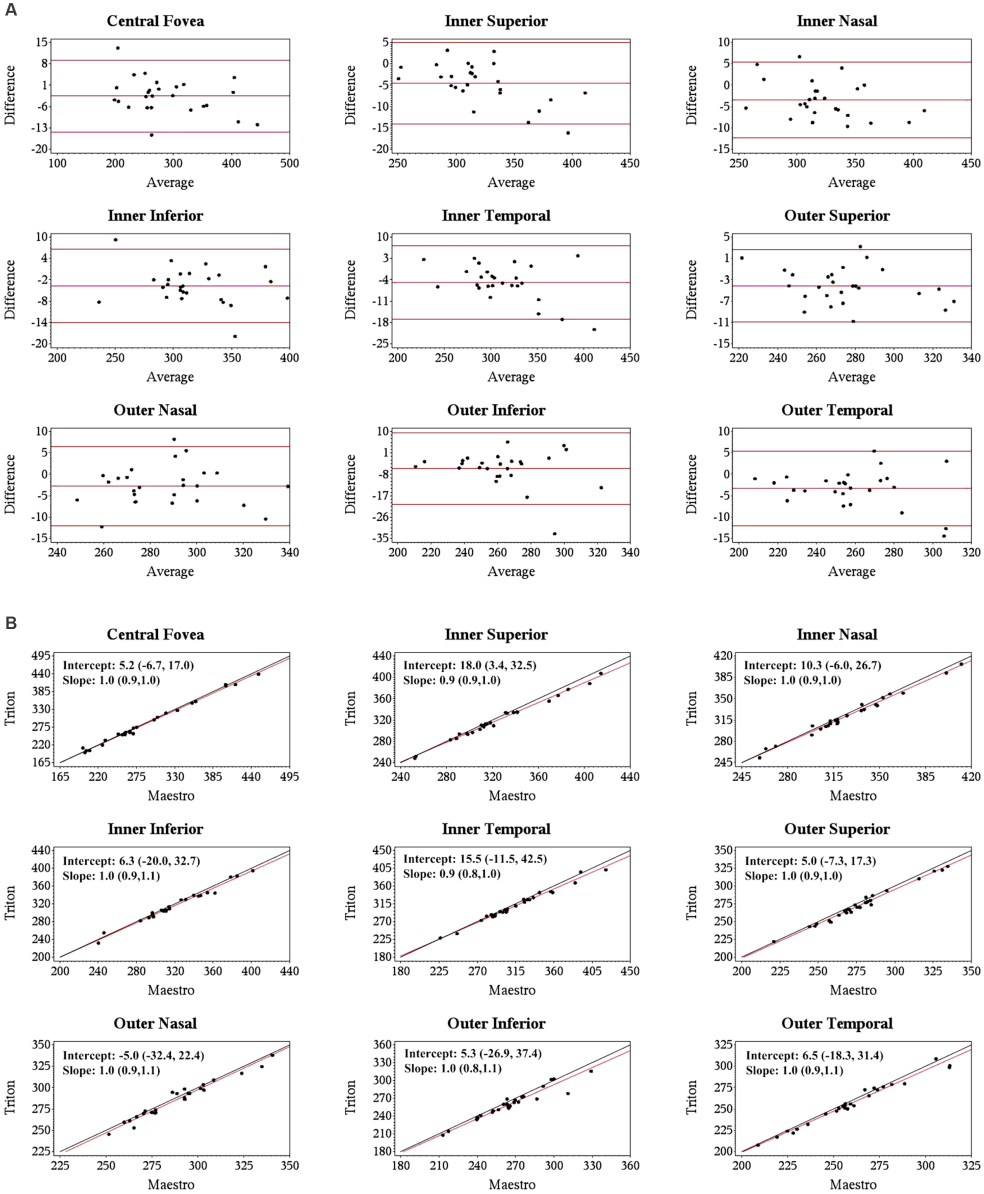
Agreement of full retina thickness measurements from the macular cube scan of Triton SS-OCT and Maestro SD-OCT in eyes with retinal disease. **(A)** Bland–Altman plots show all the average measurement differences between the two devices are less than 6 μm. **(B)** Deming regression plots of full retina thickness from the macular cube scan. The plots illustrate the fitted linear models (red line) and the identity lines (Triton measurement = Maestro measurement, slope = 1) (black line). Intercepts and slopes are shown as mean (95% confidence interval) indicate good agreement of the measurements between Triton and Maestro.

Specifically, for the thickness of different retinal layers, the mean measurements were similar from the corresponding scan types of the two devices [Triton wide scan vs. Maestro wide scan (12 mm × 9 mm for both) and Triton macular cube scan (7 mm × 7 mm) vs. Maestro macular cube scan (6 mm × 6 mm)]. Triton showed slightly lower measurements of the full retina (Table 2, absolute value of mean differences ≤5.5 μm) and GCL+ (Supplementary Table S1, absolute value of mean differences ≤2.8 μm) thickness compared to the Maestro in both healthy eyes and eyes with retinal disease. For GCL++ thickness (Table 3), Triton showed higher measurements from the wide scan and lower measurements from the macular cube scan in eyes with retinal disease. These measurement differences were small (mean difference <6 μm) and not statistically significant. Overall, excellent agreement was found across all measurements in both groups. Representative Bland–Altman plots and Deming regression plots are shown in Figures 1, 2 indicating good agreement of full retinal thickness measurements from both wide scan and macular cube scan between Triton and Maestro in eyes with retinal disease.

**Table 2:** Full retinal thickness agreement between Triton and Maestro.

	Triton 12 mm × 9 mm wide scan vs. Maestro 12 mm × 9 mm wide scan				Triton 7 mm × 7 mm macular cube scan vs. Maestro 6 mm × 6 mm macular cube scan			
	Measurements (mean ± SD)		Difference (mean ± SD)	95% LOA	Measurements (mean ± SD)		Difference (mean ± SD)	95% LOA
	Triton	Maestro			Triton	Maestro		
**Healthy group**								
Central fovea	253.3 ± 25.7	254.9 ± 27.2	−1.6 ± 5.8	−13.3, 10.0	252.8 ± 25.3	252.0 ± 27.5	0.7 ± 6.2	−11.7, 13.1
Inner superior	318.0 ± 15.0	319.8 ± 15.3	−1.7 ± 4.5	−10.7, 7.3	318.4 ± 15.5	321.1 ± 16.0	−2.7 ± 4.5	−11.7, 6.4
Inner nasal	320.1 ± 16.0	321.6 ± 15.6	−1.6 ± 4.4	−10.4, 7.3	319.9 ± 16.7	321.9 ± 16.7	−1.9 ± 4.2	−10.4, 6.6
Inner inferior	313.8 ± 16.6	315.5 ± 16.6	−1.8 ± 3.3	−8.3, 4.8	313.8 ± 17.1	315.5 ± 17.7	−1.6 ± 4.2	−10.0, 6.8
Inner temporal	304.5 ± 16.7	306.0 ± 16.4	−1.6 ± 4.2	−10.0, 6.8	304.0 ± 305.4	305.4 ± 17.3	−1.4 ± 4.4	−10.3, 7.5
Outer superior	273.7 ± 14.8	276.3 ± 16.7	−2.6 ± 3.4	−9.3, 4.2	273.3 ± 15.4	276.9 ± 16.3	−3.6 ± 3.4	−10.4, 3.3
Outer nasal	290.9 ± 18.1	292.2 ± 18.2	−1.3 ± 2.9	−7.2, 4.5	289.6 ± 18.6	291.4 ± 18.8	−1.8 ± 3.3	−8.4, 4.7
Outer inferior	263.4 ± 17.0	264.8 ± 17.0	−1.5 ± 3.0	−7.4, 4.5	263.0 ± 17.7	265.2 ± 18.3	−2.2 ± 3.6	−9.3, 5.0
Outer temporal	259.2 ± 14.1	261.3 ± 14.5	−2.1 ± 2.5	−7.2, 2.9	257.5 ± 14.4	258.7 ± 15.7	−1.3 ± 3.6	−8.4, 5.8
**Retina group**								
Central fovea	280.4 ± 65.0	284.3 ± 66.0	−3.9 ± 7.2	−18.2, 10.5	287.4 ± 69.1	289.9 ± 71.1	−2.5 ± 5.9	−14.2, 9.2
Inner superior	318.2 ± 40.0	321.5 ± 40.0	−3.3 ± 4.1	−11.6, 5.0	319.0 ± 37.7	323.6 ± 40.6	−4.6 ± 4.7	−14.0, 4.9
Inner nasal	320.3 ± 33.4	323.9 ± 34.8	−3.6 ± 4.9	−13.4, 6.1	321.0 ± 34.5	325.0 ± 36.1	−3.4 ± 4.4	−12.2, 5.4
Inner inferior	311.0 ± 35.9	313.8 ± 36.4	−2.8 ± 8.8	−20.4, 14.8	315.7 ± 36.6	319.4 ± 37.9	−3.7 ± 5.1	−14.0, 6.6
Inner temporal	304.9 ± 36.1	307.9 ± 34.6	−3.1 ± 7.0	−10.8, 4.2	310.9 ± 40.3	315.8 ± 43.2	−4.9 ± 6.0	−16.9, 7.1
Outer superior	272.5 ± 24.3	275.7 ± 25.6	−3.3 ± 3.8	−10.8, 4.2	273.3 ± 25.7	277.5 ± 26.6	−4.1 ± 3.4	−10.9, 2.6
Outer nasal	287.4 ± 21.3	290.3 ± 23.6	−2.9 ± 5.0	−13.0, 7.1	285.3 ± 22.4	288.1 ± 22.4	−2.7 ± 4.6	−12.0, 6.5
Outer inferior	258.5 ± 23.8	261.8 ± 23.8	−3.3 ± 8.3	−20.0, 13.3	260.4 ± 25.0	265.9 ± 26.7	−5.5 ± 7.5	−20.6, 9.6
Outer temporal	255.0 ± 25.0	258.1 ± 24.7	−3.2 ± 4.9	−12.9, 6.6	256.7 ± 25.9	260.0 ± 27.0	−3.3 ± 4.4	−12.0, 5.5

Unit: μm. SD, standard deviation; LOA, limit of agreement.

**Table 3:** Ganglion cell complex thickness agreement between Triton and Maestro.

	Triton 12 mm × 9 mm wide scan vs. Maestro 12 mm × 9 mm wide scan				Triton 7 mm × 7 mm macular cube scan vs. Maestro 6 mm × 6 mm macular cube scan			
	Measurements (mean ± SD)		Difference (mean ± SD)	95% LOA	Measurements (mean ± SD)		Difference (mean ± SD)	95% LOA
	Triton	Maestro			Triton	Maestro		
**Healthy group**								
Superior	107.5 ± 7.5	107.6 ± 8.2	−0.1 ± 1.8	−3.7, 3.6	107.6 ± 7.9	108.5 ± 8.0	−0.9 ± 1.4	−3.8, 2.0
Superior nasal	119.3 ± 8.8	119.4 ± 9.3	−0.1 ± 1.5	−3.1, 2.9	118.7 ± 8.7	119.4 ± 9.1	−0.7 ± 1.4	−3.5, 2.1
Superior temporal	94.6 ± 6.1	94.8 ± 6.8	−0.1 ± 1.2	−2.6, 2.3	93.9 ± 6.3	94.4 ± 6.6	−0.5 ± 1.4	−3.3, 2.4
Inferior	107.0 ± 8.8	106.72 ± 9.0	0.3 ± 1.3	−2.3, 2.9	106.7 ± 9.0	107.3 ± 9.3	−0.6 ± 1.4	−3.5, 2.2
Inferior nasal	119.7 ± 9.9	119.6 ± 10.4	0.1 ± 1.3	−2.5, 2.7	119.5 ± 10.2	120.1 ± 10.4	−0.6 ± 1.3	−3.2, 1.9
Inferior temporal	97.9 ± 7.2	98.3 ± 7.8	−0.4 ± 1.3	−3.0, 2.2	97.2 ± 7.6	97.5 ± 8.0	−0.3 ± 1.7	−3.8, 3.2
Average	107.7 ± 7.6	107.7 ± 8.1	−0.0 ± 1.2	−2.4, 2.3	107.3 ± 7.8	107.9 ± 8.1	−0.6 ± 1.1	−2.8, 1.6
**Retina group**								
Superior	112.2 ± 16.6	111.4 ± 17.3	0.8 ± 2.2	−3.6, 5.2	113.0 ± 19.7	113.8 ± 20.5	−0.8 ± 1.8	−4.4, 2.8
Superior nasal	121.5 ± 16.3	121.5 ± 7.6	0.0 ± 2.4	−4.8, 4.8	121.3 ± 17.6	121.7 ± 18.0	−0.4 ± 2.1	−4.6, 3.7
Superior temporal	97.3 ± 14.0	96.8 ± 13.6	0.4 ± 2.7	−4.9, 5.7	99.0 ± 15.4	99.5 ± 15.8	−0.5 ± 1.9	−4.3, 3.2
Inferior	108.0 ± 13.6	107.5 ± 15.1	0.5 ± 5.3	−10.1, 11.2	108.2 ± 17.2	109.5 ± 19.0	−1.3 ± 4.3	−10.0, 7.3
Inferior nasal	121.9 ± 15.4	121.0 ± 15.1	0.8 ± 2.1	−3.5, 5.1	122.7 ± 18.2	122.8 ± 20.3	−0.0 ± 3.4	−6.8, 6.8
Inferior temporal	99.5 ± 14.3	99.2 ± 15.2	0.3 ± 3.8	−7.4, 7.9	100.0 ± 17.2	100.7 ± 17.4	−0.7 ± 2.3	−5.2, 3.8
Average	110.1 ± 14.0	109.6 ± 14.2	0.5 ± 1.7	−3.0, 3.9	110.7 ± 16.2	111.4 ± 17.2	−0.7 ± 1.8	−4.3, 3.0

Unit: μm. SD, standard deviation; LOA, limit of agreement.

Overall, the repeatability and reproducibility estimates (reproducibility/repeatability limit and CV%) indicate high precision in both devices. Table 4 presents the repeatability and reproducibility for the full retinal thickness of the Healthy and Retina groups. The CV%s range between 0% and 1% for the Triton device; in comparison, Maestro had higher CV%s with a maximum of 1.8%. When comparing to the Retina group, the precision estimates were generally lower in the Healthy group with repeatability limits less than 4 μm and reproducibility limits less than 6 μm for Triton, and repeatability limits less than 10 μm and reproducibility less than 12 μm for Maestro. In general, the wide scan measurements had slightly inferior repeatability/reproducibility limits and CV%s compared with the macular cube measurements. Supplementary Table S2 and Table 5 summarize the repeatability and reproducibility estimates of GCIPL/GCL+ and GCC/GCL++ thickness measurements in Healthy and Retina groups. The repeatability and reproducibility of GCIPL/GCL+ thickness measurements were both good in Triton and Maestro (Supplementary Table S2). For both devices, all the limits were less than 7.2 μm. As expected, the repeatability and reproducibility limits of the Retina group were generally higher than that of the Healthy group. In the Healthy group, all the repeatability and reproducibility CV%s of both Triton and Maestro ranged between 0% and 1% with only 1 exception. In the Retina group, the range of repeatability CV%s of wide scan was 0.8% to 2.6% for the Triton and 1.1% to 3.3% for the Maestro; and the range of repeatability CV%s of macular cube scan was 0.7% to 1.9% for the Triton and also 0.7% to 1.9% for the Maestro. The same trends were found for the reproducibility CV%s.

**Table 4:** Repeatability and reproducibility of full retina thickness measurements.

	Triton 12 mm × 9 mm wide scan			Maestro 12 mm × 9 mm wide scan			Triton 7 mm × 7mm macular cube scan			Maestro 6 mm × 6 mm macular cube scan		
	SD	Limit	CV%	SD	Limit	CV%	SD	Limit	CV%	SD	Limit	CV%
**Repeatability**												
*Healthy group*												
Central fovea	1.3	3.7	0.5	3.5	9.8	1.4	1.0	2.8	0.4	2.1	5.8	0.8
Inner superior	0.9	2.5	0.3	1.8	5.1	0.6	0.8	2.3	0.3	1.7	4.9	0.5
Inner nasal	0.7	1.8	0.2	1.8	5.0	0.6	1.0	2.8	0.3	1.5	4.3	0.5
Inner inferior	0.7	2.0	0.2	2.4	6.7	0.8	1.0	2.7	0.3	1.7	4.7	0.5
Inner temporal	0.7	1.9	0.2	1.8	5.0	0.6	1.0	2.8	0.3	1.6	4.5	0.5
Outer superior	1.1	3.0	0.4	1.5	4.1	0.5	1.0	2.7	0.4	1.6	4.4	0.6
Outer nasal	0.7	1.9	0.2	1.1	3.0	0.5	0.6	1.7	0.2	1.1	3.0	0.4
Outer inferior	0.8	2.3	0.3	1.2	3.5	0.5	1.0	2.8	0.4	1.1	3.1	0.4
Outer temporal	0.7	2.0	0.3	1.0	2.9	0.4	1.0	2.8	0.4	1.2	3.4	0.5
*Retina group*												
Central fovea	1.7	4.9	0.6	4.6	12.7	1.6	2.3	6.5	0.8	4.0	11.1	1.4
Inner superior	1.7	4.8	0.5	2.0	5.7	0.6	1.2	3.4	0.4	1.8	4.9	0.5
Inner nasal	1.1	3.1	0.3	2.2	6.2	0.7	1.5	4.2	0.5	1.7	4.8	0.5
Inner inferior	2.1	5.8	0.7	4.2	11.7	1.3	2.7	7.6	0.9	3.6	10.0	1.1
Inner temporal	1.1	3.1	0.4	3.9	10.9	1.3	1.4	4.0	0.5	2.0	5.7	0.6
Outer superior	1.4	3.8	0.5	1.9	5.4	0.7	1.4	4.0	0.5	1.8	5.2	0.7
Outer nasal	0.9	2.4	0.3	3.2	9.0	1.1	1.6	4.4	0.6	2.0	5.5	0.7
Outer inferior	1.6	4.4	0.6	3.1	8.6	1.2	1.6	4.4	0.6	2.7	7.6	1.0
Outer temporal	1.0	2.7	0.4	2.8	7.7	1.1	1.8	5.1	0.7	2.3	6.4	0.9
**Reproducibility**												
*Healthy group*												
Central fovea	1.7	4.7	0.7	4.0	11.2	1.6	1.5	4.3	0.6	2.6	7.3	1.0
Inner superior	1.6	4.5	0.5	2.5	7.0	0.8	1.5	4.1	0.5	2.3	6.5	0.7
Inner nasal	1.5	4.2	0.5	2.6	7.2	0.8	1.8	5.1	0.6	2.2	6.2	0.7
Inner inferior	1.5	4.1	0.5	3.1	8.6	1.0	1.8	4.9	0.6	2.3	6.6	0.7
Inner temporal	1.4	4.1	0.5	2.6	7.3	0.9	1.9	5.3	0.6	2.0	5.6	0.7
Outer superior	1.6	4.4	0.6	2.0	5.5	0.7	1.5	4.3	0.6	2.3	6.6	0.8
Outer nasal	1.5	4.1	0.5	1.7	4.7	0.6	1.4	3.9	0.5	1.6	4.6	0.6
Outer inferior	1.4	4.0	0.5	1.8	4.9	0.7	1.7	4.8	0.6	1.8	4.9	0.7
Outer temporal	1.4	3.9	0.5	1.5	4.2	0.6	1.7	4.6	0.6	1.8	5.1	0.7
*Retinal group*												
Central fovea	3.3	9.2	1.2	5.1	14.4	1.8	2.5	7.0	0.9	4.4	12.3	1.5
Inner superior	2.2	6.3	0.7	2.4	6.8	0.8	2.7	7.5	0.8	2.5	7.1	0.8
Inner nasal	2.5	7.1	0.8	2.9	8.2	0.9	2.4	6.6	0.7	2.8	7.8	0.9
Inner inferior	3.0	8.3	1.0	4.7	13.1	1.5	2.9	8.3	0.9	4.5	12.7	1.4
Inner temporal	2.0	5.6	0.7	4.7	13.2	1.5	2.3	6.5	0.8	3.2	9.1	1.0
Outer superior	2.3	6.6	0.9	2.9	8.1	1.0	2.1	5.8	0.8	2.6	7.2	0.9
Outer nasal	1.7	4.7	0.6	3.3	9.2	1.1	2.0	5.7	0.7	2.6	7.3	0.9
Outer inferior	2.4	6.8	0.9	3.9	11.0	1.5	2.0	5.5	0.8	4.0	11.1	1.5
Outer temporal	1.8	5.1	0.7	3.1	8.7	1.2	2.1	6.0	0.8	2.6	7.3	1.0

**Table 5:** Repeatability and reproducibility of ganglion cell complex thickness measurements.

	Triton 12 mm × 9 mm wide scan			Maestro 12 mm × 9 mm wide scan			Triton 7 mm × 7 mm macular cube scan			Maestro 6 mm × 6 mm macular cube scan		
	SD	Limit	CV%	SD	Limit	CV%	SD	Limit	CV%	SD	Limit	CV%
**Repeatability**												
*Healthy group*												
Superior	0.7	2.1	0.7	0.8	2.1	0.7	0.7	1.9	0.6	0.6	1.8	0.6
Superior nasal	0.6	1.7	0.5	0.7	1.9	0.6	0.5	1.4	0.4	0.6	1.7	0.5
Superior temporal	0.6	1.6	0.6	0.5	1.5	0.5	0.7	2.0	0.8	0.6	1.8	0.7
Inferior	0.6	1.7	0.6	0.7	1.9	0.6	0.7	1.9	0.6	0.7	1.9	0.6
Inferior nasal	0.5	1.5	0.5	0.7	1.9	0.6	0.7	1.8	0.6	0.6	1.7	0.5
Inferior temporal	0.5	1.5	0.6	0.6	1.7	0.6	0.7	2.1	0.8	0.7	1.9	0.7
Average	0.4	1.2	0.4	0.5	1.3	0.4	0.5	1.3	0.4	0.4	1.2	0.4
*Retina group*												
Superior	1.2	3.3	1.1	3.3	9.3	3.0	1.3	3.5	1.1	1.2	3.3	1.0
Superior nasal	1.0	2.7	0.8	1.7	4.6	1.4	1.1	2.9	0.9	0.9	2.7	0.8
Superior temporal	0.8	2.2	0.8	1.6	4.4	1.6	1.0	2.9	1.1	1.0	2.9	1.0
Inferior	1.0	2.8	0.9	2.4	6.6	2.2	1.1	3.1	1.0	1.9	5.4	1.8
Inferior nasal	0.9	2.4	0.7	1.4	3.8	1.1	1.4	3.9	1.1	1.3	3.5	1.0
Inferior temporal	0.8	2.2	0.8	1.7	4.8	1.7	1.0	2.8	1.0	1.0	2.7	0.9
Average	0.6	1.6	0.5	1.1	3.1	1.0	0.9	2.5	0.8	0.7	2.1	0.7
**Reproducibility**												
*Healthy group*												
Superior	0.9	2.4	0.8	1.0	2.9	1.0	0.9	2.4	0.8	0.8	2.3	0.8
Superior nasal	0.8	2.3	0.7	1.0	2.9	0.9	0.7	1.9	0.6	0.8	2.2	0.7
Superior temporal	0.8	2.2	0.8	0.7	1.9	0.7	0.9	2.6	1.0	0.8	2.3	0.9
Inferior	0.8	2.2	0.7	0.8	2.3	0.8	0.9	2.6	0.9	0.8	2.3	0.8
Inferior nasal	0.8	2.2	0.7	0.8	2.4	0.7	0.8	2.2	0.7	0.8	2.3	0.7
Inferior temporal	0.8	2.3	0.8	0.7	2.0	0.7	1.0	2.8	1.0	0.9	2.6	1.0
Average	0.6	1.8	0.6	0.6	1.7	0.6	0.7	1.8	0.6	0.6	1.6	0.5
*Retinal group*												
Superior	1.6	4.5	1.5	3.3	9.4	3.0	1.4	4.0	1.3	1.2	3.5	1.1
Superior nasal	1.3	3.6	1.1	1.7	4.7	1.4	1.2	3.4	1.0	1.2	3.2	0.9
Superior temporal	1.1	3.1	1.2	1.7	4.6	1.7	1.2	3.4	1.2	1.3	3.6	1.3
Inferior	1.3	3.7	1.2	2.4	6.8	2.3	1.3	3.5	1.2	2.3	6.3	2.1
Inferior nasal	1.1	3.2	0.9	1.5	4.3	1.3	1.5	4.1	1.2	1.4	3.8	1.1
Inferior temporal	1.2	3.3	1.2	1.9	5.5	2.0	1.1	3.2	1.1	1.5	4.1	1.4
Average	0.9	2.7	0.9	1.1	3.2	1.0	1.0	2.8	0.9	0.9	2.4	0.8

Similarly, the repeatability and reproducibility of GCC/GCL++ thickness measurements were both good and mostly comparable between the two devices (Table 5). All the limits of repeatability and reproducibility were less than 10 μm, and the limits of the Retina group were higher. In the Healthy group, all the repeatability and reproducibility CV%s of both Triton and Maestro were ≤1%; CV%s of the Retina group were higher-the maximum repeatability and reproducibility CV%s for the Triton were 1.1% and 1.5% for the wide scan, and 1.1% and 1.3% for the macular cube scan, respectively; and for the Maestro, the maximum repeatability and reproducibility CV%s were 3.0% and 3.0% for the wide scan, and 1.8% and 2.1% for the macular cube scan.

## Discussion

This study found excellent agreement and good repeatability and reproducibility of the full retina, GCIPL/GCL+, and GCC/GCL++ thickness measurements obtained using the Triton SS-OCT and Maestro SD-OCT in healthy eyes and eyes with retinal disease.

Establishing the margins of repeatability and reproducibility for OCT-based measurements is important to support use of such metrics in clinical practice and in clinical studies is important. The accurate measurement of retinal layers has a particularly important role in the diagnosis and management of retinal diseases. Studying repeatability enables an identification of genuine clinical change from naturally occurring measurement variability. Clinicians can then define a threshold to recognize when a true change in a condition has occurred, which may then be employed in clinical practice to decide whether further treatment is necessary or to identify a therapeutic response (14, 15). Reproducibility is relevant to multicenter clinical trials because measurements acquired at multiple sites with various operators and devices will be assessed, but it is also relevant at clinics that have multiple devices and operators, or practices where a patient may visit satellite locations (13). Because SS-OCT and SD-OCT are the two main commercially available OCT systems in retinal practices, it is crucial to understand if they can provide reliable measurements and to evaluate the underlying interchangeability.

Several studies have investigated the agreement of thickness measurements between SS-OCT and SD-OCT. Lee et al. (16) evaluated agreement of the measurements from the macular cube scan between SS-OCT (DRI-OCT, Topcon) and SD-OCT (Cirrus HD-OCT, Carl Zeiss Meditec) in healthy eyes, and found that SD-OCT yielded larger GCIPL thickness values than SS-OCT, which is consistent with the current study finding that Maestro showed higher thickness measurements. This is expected because a smaller measurement region (Triton 7 mm × 7 mm vs. Maestro 6 mm × 6 mm) contains a relatively larger proportion of thicker retinal areas (16). However, contrary to this study, the study by Lee et al. (16) showed that the differences in thickness measurements between the DRI-OCT and Cirrus HD-OCT were statistically significant, with a mean difference that was approximately 5 fold larger than the current study. This discrepancy could be mainly due to the obvious different measurement regions in their study, which includes a 6 mm diameter circle for the DRI-OCT and a 4.8 mm × 4 mm elliptical annulus for Cirrus. This assumption has been confirmed by a study by Yang et al. (17). Yang et al. (17) found excellent agreement of GCIPL thickness between SS-OCT wide scan (DRI-OCT, Topcon) and SD-OCT macular cube scan (Cirrus HD-OCT, Carl Zeiss Meditec) for healthy eyes using identical measurement regions and segmentations between the two devices (17). To sum these studies up, agreement of OCT measurements mainly depends on the segmentation algorithm (18) and the measurement region/grid. Retinal pathology adversely affects the agreement, consistent with findings from this study, in which eyes with retinal disease had larger measurement differences between the Triton and Maestro.

Despite the good repeatability and reproducibility of both devices in our study, we found several factors that appeared to affect retinal thickness measurement precision. First, as expected and consistent with other studies (13), the repeatability and reproducibility in eyes with retinal disease were worse compared with healthy eyes. We postulate that retinal pathology in these eyes may have contributed to higher variability of measurements in this cohort by affecting foveal detection, grid positioning, and segmentation. However, the repeatability and reproducibility within this cohort remained good, with repeatability CV%s of less than 2.6% for Triton and less than 3.4% for Maestro; and reproducibility CV%s less than 3.3% for Triton and less than 3.5% for Maestro. Second, in this study, despite Triton SS-OCT generally having lower precision estimates than the Maestro SD-OCT, particularly in eyes with retinal disease, both devices provided reliable measurements with high repeatability and reproducibility. Studies to compare precision of retinal thickness measurements of SS-OCT and SD-OCT are limited, especially in eyes with retinal disease. One study by Lee et al. (16) reported similar repeatability of GCIPL thickness measurement between SS-OCT (DRI-OCT, Topcon) and SD-OCT (Cirrus HD-OCT, Carl Zeiss Meditec) in healthy eyes (16). Image quality has a positive correlation with OCT-based measurements and is an essential factor affecting measurement reliability (19–23). In Lee’s study (16), the Cirrus HD-OCT and DRI-OCT devices had similar rates of poor-quality scans. In the current study, all included images had acceptable quality that may have minimized quality variations, thereby facilitating good precision. This indicates the potential benefit of primary quality control by operators in real-world setting. Similar precision of measurements from Triton SS-OCT and Maestro SD-OCT are expected given the fact that they have only minor differences in axial resolution and pixel calibration factor, and they use similar software and algorithms.

We also found that scan type had an influence on the measurement precision, although the direction of the difference varied across parameters. For full retinal thickness, the wide scan measurements were slightly less repeatable and reproducible compared with macular cube scan measurements; for GCIPL/GCL+ and GCC/GCL++ thickness, precision of wide and macular cube scans were mostly comparable; Triton wide scan had better repeatability and reproducibility in comparison with Maestro wide scan, while for the macular cube scan, the precision of the two devices was mostly comparable. The wide scan type covers both the macular and circumpapillary areas, and has several advantages over the macular cube scan, including reducing imaging time, minimizing alignment errors, and reducing fixation errors (24, 25). However, the advantages of minimizing alignment errors and reducing fixation errors likely did not contribute to the results of this study, because such errors may have been filtered out by allowing technicians to scan subjects multiple times in order to acquire an image with acceptable quality as defined by each device manufacturer’s user instructions. The expected better precision results of wide scans are likely to be seen in a real-world setting. Application of wide scan in clinical practice has been suggested in glaucoma management (24, 26). Given that retinal pathologies often go beyond the macula and are not isolated to the relatively small region of the macular cube scan, adoption of a wide scan into clinical practice may provide significant benefits in the imaging of retinal disorders and has been used for detection of various retina diseases (26–28). However, evaluation of wide scan measurement precision is lacking in eyes with retinal disease. This study found high precision of wide scan measurements of GCIPL/GCL+ and GCC/GCL++ thickness but lower precision in full retinal thickness. For instance, reproducibility limits of average GCIPL and GCC thickness from the wide scan were only 2.0 μm and 2.7 μm for Triton, and 2.3 μm and 3.2 μm for Maestro. However, in terms of full retinal thickness, the reproducibility limits across sectors were higher; <9.2 μm for Triton and <14.4 μm for Maestro. The underlying rationale is unclear, but these results highlight the consideration of consistent device and scan type between patients or over time in the same patient when full retinal thickness is of interest.

Our study has several limitations. First, with 25 eyes for the Healthy group and 26 eyes for the Retina group, the sample size for this study is comparatively modest. The ANOVA model and the 95% LOA were used to estimate the sample size, and 21 eyes per group were judged suitable. For the Retina group, this study included a variety of different retina diseases with relatively few eyes having one specific disease, thus, we were unable to determine whether the type of disease had an impact on repeatability and reproducibility. Future research on this topic may be of interest. Second, there was a difference in age between the cohort with retinal disease and the healthy cohort. However, unlike RNFL thickness, age is not a limiting factor in achieving reliable retinal thickness (29). Besides, all estimates were calculated by cohort without inter-group comparison. Yet, this variation should be taken into consideration when interpreting the results.

## Conclusion

In conclusion, this study demonstrated excellent agreement of retinal thickness measurements between the Triton SS-OCT and Maestro SD-OCT, as well as high precision of all measurements obtained using the two devices, in healthy eyes and eyes with retinal disease. These findings strongly support the use of Triton SS-OCT and Maestro SD-OCT to provide reliable and interchangeable test results of retinal layer measurements in clinical practice.

## Data availability statement

The raw data supporting the conclusions of this article will be made available by the authors, without undue reservation.

## Ethics statement

The studies involving humans were approved by IntegReview Institutional Review Board. The studies were conducted in accordance with the local legislation and institutional requirements. The participants provided their written informed consent to participate in this study.

## Author contributions

HH: Conceptualization, Data curation, Methodology, Validation, Visualization, Writing – original draft, Writing – review & editing. MD: Conceptualization, Data curation, Formal analysis, Methodology, Supervision, Validation, Visualization, Writing – original draft, Writing – review & editing. NE-N: Visualization, Writing – original draft, Writing – review & editing. JF: Data curation, Investigation, Methodology, Writing – review & editing. SS: Conceptualization, Visualization, Writing – review & editing.
