# Supplementary material for: Agreement, repeatability, and reproducibility of quantitative retinal layer assessment using swept-source and spectral-domain optical coherence tomography in eyes with retinal diseases
Source: Front Med (Lausanne). 2023 Dec 18;10:1281751. doi: 10.3389/fmed.2023.1281751 (PMC10757970; doi:10.3389/fmed.2023.1281751)
Supplement: Supplementary file 1 [file Table_1.DOCX]

**Supplementary Table 1. Ganglion Cell and Internal Plexiform Layer Thickness Agreement Between Triton and Maestro**

|  | **Triton 12mm×9mm Wide Scan vs. Maestro 12mm×9mm Wide Scan** | | | | **Triton 7mmx7mm Macular Cube Scan vs. Maestro 6mmx6mm Macular Cube Scan** | | | |
| --- | --- | --- | --- | --- | --- | --- | --- | --- |
|  | **Measurements (Mean±SD)** | | **Difference (Mean±SD)** | **95% LOA** | **Measurements (Mean±SD)** | | **Difference (Mean±SD)** | **95% LOA** |
|  | **Triton** | **Maestro** |  |  | **Triton** | **Maestro** |  |  |
| **Healthy group** | | | | | | | | |
| Superior | 70.4±5.6 | 72.2±6.0 | -1.8±1.0 | -3.8, 0.2 | 69.8±5.7 | 72.6±6.1 | -2.8±1.5 | -5.8, 0.2 |
| Superior Nasal | 74.6±6.6 | 76.4±6.9 | -1.8±1.0 | -3.7, 0.1 | 74.5±6.4 | 76.5±6.8 | -2.0±1.1 | -4.3, 0.2 |
| Superior Temporal | 70.9±5.4 | 72.4±5.8 | -1.4±0.9 | -3.2, 0.3 | 71.4±4.8 | 72.6±6.1 | -1.2±1.2 | -3.7, 1.3 |
| Inferior | 67.7±6.3 | 69.4±6.2 | -1.8±1.2 | -4.1, 0.6 | 67.2±6.2 | 69.7±6.4 | -2.5±1.2 | -5.0, -0.0 |
| Inferior Nasal | 73.2±6.5 | 75.1±6.7 | -1.9±1.0 | -3.8, 0.0 | 72.9±6.8 | 75.0±6.5 | -2.1±0.8 | -3.8, -0.4 |
| Inferior Temporal | 72.1±6.5 | 74.0±6.8 | -1.9±1.0 | -3.9, 0.1 | 72.6±6.4 | 73.9±6.9 | -1.3±1.2 | -3.8, 1.1 |
| Average | 71.5±5.9 | 73.3±6.1 | -1.8±0.6 | -2.9, -0.6 | 71.4±5.8 | 73.4±6.0 | -2.0±0.8 | -3.6, -0.4 |
| **Retina group** | | | | | | | | |
| Superior | 67.8±6.8 | 69.3±7.1 | -1.5±2.8 | -7.0, 4.1 | 66.5±6.8 | 68.7±7.6 | -2.3±1.8 | -5.8, 1.2 |
| Superior Nasal | 72.0±7.2 | 74.4±9.5 | -2.3±4.6 | -11.5, 6.8 | 69.6±37.7 | 71.6±6.7 | -1.9±1.8 | -5.5, 1.7 |
| Superior Temporal | 69.4±10.3 | 70.5±9.5 | -1.1±2.3 | -5.6, 3.4 | 72.5±9.9 | 73.0±10.5 | -0.5±3.3 | -7.1, 6.0 |
| Inferior | 64.2±8.3 | 65.5±8.9 | -1.2±4.2 | -9.7, 7.2 | 64.3±8.4 | 67.0±8.4 | -2.7±2.9 | -8.4, 3.1 |
| Inferior Nasal | 71.3±7.7 | 72.9±8.8 | -1.6±2.2 | -6.1, 2.9 | 69.7±7.3 | 71.4±8.2 | -1.7±2.0 | -5.8, 2.3 |
| Inferior Temporal | 70.8±10.8 | 72.2±11.1 | -1.4±3.1 | -7.7, 4.9 | 73.4±12.1 | 74.3±12.2 | -0.9±2.1 | -5.0, 3.2 |
| Average | 69.3±7.7 | 70.8±8.0 | -1.5±1.0 | -3.5, 0.5 | 69.3±7.6 | 71.0±8.0 | -1.7±1.2 | -4.0, 0.7 |
| Unit: µm. Abbreviations: SD, standard deviation; LOA, limit of agreement. | | | | | | | | |
