# Supplementary material for: Agreement, repeatability, and reproducibility of quantitative retinal layer assessment using swept-source and spectral-domain optical coherence tomography in eyes with retinal diseases
Source: Front Med (Lausanne). 2023 Dec 18;10:1281751. doi: 10.3389/fmed.2023.1281751 (PMC10757970; doi:10.3389/fmed.2023.1281751)
Supplement: Supplementary file 2 [file Table_2.DOCX]

**Supplementary Table 2. Repeatability and Reproducibility of Ganglion Cell and Internal Plexiform Layer Thickness Measurements**

|  | **Triton 12mm×9mm Wide Scan** | | | **Maestro12mm×9mm Wide Scan** | | | **Triton 7mmx7mm Macular Cube Scan** | | | **Maestro 6mmx6mm Macular Cube Scan** | | |
| --- | --- | --- | --- | --- | --- | --- | --- | --- | --- | --- | --- | --- |
|  | **SD** | **Limit** | **CV%** | **SD** | **Limit** | **CV%** | **SD** | **Limit** | **CV%** | **SD** | **Limit** | **CV%** |
| **Repeatability** | | | | | | | | | | | | |
| **Healthy group** | | | | | | | | | | | | |
| Superior | 0.5 | 1.5 | 0.8 | 0.6 | 1.8 | 0.9 | 0.4 | 1.2 | 0.6 | 0.5 | 1.5 | 0.7 |
| Superior Nasal | 0.4 | 1.1 | 0.5 | 0.5 | 1.4 | 0.7 | 0.4 | 1.1 | 0.5 | 0.5 | 1.3 | 0.6 |
| Superior Temporal | 0.5 | 1.4 | 0.7 | 0.5 | 1.4 | 0.7 | 0.6 | 1.7 | 0.8 | 0.6 | 1.6 | 0.8 |
| Inferior | 0.4 | 1.1 | 0.6 | 0.6 | 1.6 | 0.8 | 0.5 | 1.3 | 0.7 | 0.5 | 1.4 | 0.7 |
| Inferior Nasal | 0.4 | 1.0 | 0.5 | 0.6 | 1.6 | 0.8 | 0.5 | 1.3 | 0.6 | 0.4 | 1.2 | 0.6 |
| Inferior Temporal | 0.5 | 1.4 | 0.7 | 0.5 | 1.5 | 0.7 | 0.7 | 1.8 | 0.9 | 0.5 | 1.4 | 0.7 |
| Average | 0.3 | 0.7 | 0.4 | 0.2 | 0.6 | 0.3 | 0.2 | 0.6 | 0.3 | 0.3 | 0.7 | 0.3 |
| **Retina group** | | | | | | | | | | | | |
| Superior | 1.4 | 3.8 | 2.0 | 1.7 | 4.8 | 2.5 | 1.0 | 2.8 | 1.5 | 1.0 | 2.8 | 1.5 |
| Superior Nasal | 1.1 | 3.0 | 1.5 | 2.5 | 6.9 | 3.3 | 1.0 | 2.9 | 1.5 | 1.2 | 3.4 | 1.7 |
| Superior Temporal | 1.8 | 5.1 | 2.6 | 2.4 | 6.7 | 3.4 | 1.4 | 3.8 | 1.9 | 1.3 | 3.7 | 1.8 |
| Inferior | 1.1 | 3.0 | 1.7 | 1.9 | 5.2 | 2.8 | 1.0 | 2.7 | 1.5 | 1.3 | 3.6 | 1.9 |
| Inferior Nasal | 1.0 | 2.9 | 1.4 | 1.4 | 4.0 | 2.0 | 1.0 | 3.0 | 1.5 | 1.2 | 3.3 | 1.6 |
| Inferior Temporal | 1.3 | 3.6 | 1.8 | 1.6 | 4.6 | 2.3 | 0.8 | 2.3 | 1.1 | 1.2 | 3.4 | 1.6 |
| Average | 0.5 | 1.5 | 0.8 | 0.8 | 2.2 | 1.1 | 0.5 | 1.4 | 0.7 | 0.5 | 1.5 | 0.7 |
| **Reproducibility** | | | | | | | | | | | | |
| **Healthy group** | | | | | | | | | | | | |
| Superior | 0.7 | 1.9 | 1.0 | 0.7 | 2.0 | 1.0 | 0.6 | 1.7 | 0.9 | 0.6 | 1.7 | 0.8 |
| Superior Nasal | 0.5 | 1.4 | 0.7 | 0.6 | 1.7 | 0.8 | 0.5 | 1.4 | 0.7 | 0.5 | 1.5 | 0.7 |
| Superior Temporal | 0.6 | 1.7 | 0.8 | 0.6 | 1.7 | 0.8 | 0.7 | 2.0 | 1.0 | 0.6 | 1.7 | 0.9 |
| Inferior | 0.5 | 1.3 | 0.7 | 0.8 | 2.1 | 1.1 | 0.6 | 1.7 | 0.9 | 0.7 | 2.0 | 1.0 |
| Inferior Nasal | 0.4 | 1.2 | 0.6 | 0.7 | 1.9 | 0.9 | 0.6 | 1.7 | 0.8 | 0.6 | 1.6 | 0.8 |
| Inferior Temporal | 0.6 | 1.6 | 0.8 | 0.6 | 1.7 | 0.8 | 0.8 | 2.1 | 1.0 | 0.6 | 1.8 | 0.8 |
| Average | 0.3 | 1.0 | 0.5 | 0.3 | 0.9 | 0.5 | 0.4 | 1.1 | 0.5 | 0.4 | 1.1 | 0.5 |
| **Retinal group** | | | | | | | | | | | | |
| Superior | 1.6 | 4.5 | 2.4 | 2.0 | 5.7 | 2.9 | 1.3 | 3.5 | 1.9 | 1.4 | 3.9 | 2.1 |
| Superior Nasal | 1.1 | 3.1 | 1.5 | 2.6 | 7.2 | 3.5 | 1.2 | 3.4 | 1.7 | 1.6 | 4.4 | 2.2 |
| Superior Temporal | 2.2 | 6.3 | 3.3 | 2.4 | 6.7 | 3.4 | 1.7 | 4.6 | 2.3 | 1.7 | 4.8 | 2.4 |
| Inferior | 1.3 | 3.7 | 2.1 | 2.1 | 5.9 | 3.2 | 1.1 | 3.0 | 1.7 | 1.9 | 5.4 | 2.9 |
| Inferior Nasal | 1.2 | 3.5 | 1.7 | 1.5 | 4.3 | 2.1 | 1.1 | 3.1 | 1.6 | 1.4 | 4.0 | 2.0 |
| Inferior Temporal | 1.4 | 3.8 | 1.9 | 2.2 | 6.0 | 3.0 | 1.1 | 3.0 | 1.5 | 1.5 | 4.2 | 2.0 |
| Average | 0.7 | 2.0 | 1.0 | 0.8 | 2.3 | 1.2 | 0.6 | 1.6 | 0.8 | 0.6 | 1.8 | 0.9 |
